# Ptomaines and Leucomaines

**Published:** 1886-03

**Authors:** F. R. Campbell


### Translations.

### Ptomaines and Leucomaines.

Translated from *L'Abeille Medicale*, January 18th, by F. R. Campbell, M. D.

M. Gautier, in a recent communication to the French Academy of Sciences (January 12, 1886), gives the results of experiments, showing the physiological action of the post mortem alkaloids. The alkaloids were obtained from putrid meat. He first experimented with—

1. *Ptomaines Extracted by Ether.*—One gramme of aqueous solution of this ptomaine injected subcutaneously in a medium sized dog produced, after twenty minutes, the following phenomena: Irregular oblique pupil, convulsive trembling, frequent pulse, a remarkable congestion of the capillaries of the tip of the ear, with two degrees increase of temperature in the part. Then the animal became stupefied and the pupil contracted. Forty minutes after the injection, spasmodic contractions of the muscles of the face and limbs and slow respiration appeared. Forty-five minutes after the administration of the ptomaine, death. The right heart was found full of clotted blood, the left was contracted and empty. The same effects were observed in frogs.

2. *Ptomaines Extracted by Chloroform.*—After the putrid meat had been exhausted by ether, chloroform was passed through it. The residue left after the evaporation of the chloroform was dissolved in acidulated water and injected hypodermically into a dog. Immediately the respiration dropped from 194 to 130 per minute, pulse slightly increased, congestion of vessels of concha of ear. In fifty minutes the effects of the ptomaine passed away. In frogs, the substance produced weakness of muscles, followed by extreme flaccidity. Respiration diminished, sensibility unimpaired, but motor nerves were partially paralyzed.

3. *Ptomaines Extracted by Amylic Alcohol.*—The residue which had been exhausted by ether and alcohol was digested with amylic alcohol, and a yellowish, slightly fluorescent, solution obtained. The ptomaines thus obtained were treated with acidulated water and injected in frogs. Immediately the pupils were dilated, cutaneous sensibility entirely disappeared, followed by general relaxation of muscles and death.

Gautier concludes, from his experiments, that the cadaveric alkaloids are highly poisonous, and that the free ptomaines are more poisonous than their salts. Mixed ptomaines injected into frogs produce the following phenomena:

1. Dilation of the pupil.
2. Tetanic convulsions, followed by flaccidity of the muscles.
3. Slowing of heart beats, rarely acceleration.
4. Entire loss of cutaneous sensibility.
5. Loss of muscular contractility.

In dogs the principal phenomena observed are:

1. Irregular and contracted pupil.
2. Marked injection of vessels of concha of ear, from paralysis of vaso-motors.
3. Greatly retarded respiration.
4. Somnolence, followed by convulsions and death.
5. Loss of muscular contractility.

The loss of muscular contractility resembles that produced by the alkaloid obtained from "toad stools," *muscarine*, but differs in its effects from *curare*. An alkaloid, with a base corresponding to the formulæ $C^8, H^{13}, N$, hydrocallidine was obtained, which was as deadly in its effects as the poison of the *cobra capello.* One milligramme of this substance will, in a few minutes, produce complete paralysis of the spinal nerves and death.

The writer then discussed the medico-legal aspects of his subject, showing how these cadaveric poisons might be mistaken for vegetable alkaloids, and pointing out means of differentiation.

*Physiological alkaloids*, or those which are normally present in the tissues and secretions, were next discussed. Gautier has denominated these substances, *leucomaines*, in contradistinction to the post mortem alkaloids which are called *ptomaines*. He first experimented with serpent poisons, and extracted various alkaloids therefrom, having a variety of effects upon the living body. None, however, were as poisonous as the original venom. Gautier then obtained similar alkaloids from human saliva, blood, urine, albumen, and especially from muscular tissues. These leucomaines accumulate in excessive quantities in the blood whenever their elimination by the skin, kidneys and intestinal tube are not sufficiently active. It is then that they affect the nervous centers, giving rise to various series of pathological phenomena which, collectively, form the pictures of diseases. This continual auto-infection is resisted in two ways—

1. By eliminating the poisons. Excretion by the kidneys is easily demonstrated. Gautier has always found leucomaines in normal urine, and confirms the statement of Bouchard, who claims that these alkaloids found in the urine are greatly increased in the course of infectious diseases, especially typhoid fever and certain cerebral diseases. Elimination by the digestive tube is also highly probable, but not so easily demonstrated, since some of the alkaloids found in fæces are undoubtedly the products of bacterial fermentation of the contents. Leucomaines are also eliminated by the skin and lungs.

2. Combustion by the oxygen of the blood. The great majority of the leucomaines are very easily oxydized, and converted into harmless compounds. The muscular alkaloids, in particular, are consumed in the blood, so that very few appear in the urine. But when, from any cause, the amount of oxygen in the blood is reduced, as in anæmia or chlorosis, these alkaloids immediately increase.

